# GraphPath: a graph attention model for molecular stratification with interpretability based on the pathway–pathway interaction network

**DOI:** 10.1093/bioinformatics/btae165

**Published:** 2024-03-26

**Authors:** Teng Ma, Jianxin Wang

**Affiliations:** Hunan Provincial Key Lab on Bioinformatics, School of Computer Science and Engineering, Central South University, Changsha 41083, Hunan, China; Hunan Provincial Key Lab on Bioinformatics, School of Computer Science and Engineering, Central South University, Changsha 41083, Hunan, China

## Abstract

**Motivation:**

Studying the molecular heterogeneity of cancer is essential for achieving personalized therapy. At the same time, understanding the biological processes that drive cancer development can lead to the identification of valuable therapeutic targets. Therefore, achieving accurate and interpretable clinical predictions requires paramount attention to thoroughly characterizing patients at both the molecular and biological pathway levels.

**Results:**

Here, we present GraphPath, a biological knowledge-driven graph neural network with multi-head self-attention mechanism that implements the pathway–pathway interaction network. We train GraphPath to classify the cancer status of patients with prostate cancer based on their multi-omics profiling. Experiment results show that our method outperforms P-NET and other baseline methods. Besides, two external cohorts are used to validate that the model can be generalized to unseen samples with adequate predictive performance. We reduce the dimensionality of latent pathway embeddings and visualize corresponding classes to further demonstrate the optimal performance of the model. Additionally, since GraphPath’s predictions are interpretable, we identify target cancer-associated pathways that significantly contribute to the model’s predictions. Such a robust and interpretable model has the potential to greatly enhance our understanding of cancer’s biological mechanisms and accelerate the development of targeted therapies.

**Availability and implementation:**

https://github.com/amazingma/GraphPath.

## 1 Introduction

Malignant tumors, commonly referred to as cancer, are a group of diseases in which cells divide continuously and excessively, leading to a major global public health problem (Fitzmaurice *et al.* 2018, Sung *et al.* 2021, Xia *et al.* 2022). Molecular heterogeneity is one of the most important concerns in cancer, meaning that during the growth and proliferation of the tumor cell, the daughter cells exhibit differences at the molecular level, resulting in variations in tumor growth rate, invasion and metastasis patterns, drug sensitivity, and other aspects (Dagogo-Jack and Shaw 2018, Sharma *et al.* 2019, Jiao *et al.* 2020, Vitale *et al.* 2021, Shi *et al.* 2023). These heterogeneities within the same type of cancer give rise to varying treatment responses and overall prognosis among patients, posing significant challenges in cancer diagnosis and management (Abeshouse *et al.* 2015, Ateeq *et al.* 2016, Punt *et al.* 2017, Buikhuisen *et al.* 2020). Recent advances in genomics, proteomics, and molecular pathology have led to the discovery of candidate biomarkers that hold potential clinical value for cancer subtype classification, staging, and prognosis (McGuire *et al.* 2017, Audenet *et al.* 2018, Zhao *et al.* 2019, 2020). For example, numerous studies in recent years have demonstrated that molecular subtypes can be identified in most human cancers and that different subtypes are significantly correlated with different therapeutic responses and clinical outcomes (McConkey and Choi 2018, Torres and Grippo 2018, Bozic and Wu 2020, Turajlic *et al.* 2020, Wolf *et al.* 2022). The application of molecular profiling technologies creates opportunities for personalized medicine. Projects such as The Cancer Genome Atlas (TCGA) and International Cancer Genome Consortium (ICGC) promise more accurate and precise definitions of tumor subtypes and better predictions of how particular tumor subtypes will respond to different treatments (Bailey *et al.* 2016, Chen *et al.* 2019, Sinkala *et al.* 2020, Jarnuczak *et al.* 2021).

High accuracy and interpretability are crucial for clinical prediction models that allow healthcare professionals to make informed decisions to provide personalized care and ultimately improve the quality of healthcare services. In this context, bio-inspired computing is gaining prominence, where not only the system function but also the system structure needs to be simulated to understand the mechanisms that lead to the predicted outcome. For example, DCell (Ma *et al.* 2018) models the relationship between genotype and growth rate in yeast. The structure of this model consists of first-layer nodes that represent genes and second-layer nodes that are constructed as functional groups defined hierarchically according to the Gene Ontology database. The model’s transparency assisted in explaining the relationship between mutations and growth rate. Recently, with the development of cancer research, it has become a consensus that multiple driver pathways are cooperatively involved in the transformation process from a normal cell to a tumor during cancer development (Chen and Zhang 2017). Therefore, more attention has been paid to identifying driver pathways and functional modules rather than individual genes, leading to the emergence of pathway-based deep neural networks. A pathway represents a specific biological process, which is a series of biochemical reaction steps that lead to specific products or changes in a cell. The idea behind this modeling approach is that genes and their products function together in a pathway module, rather than working independently of each other. Additionally, while cancer genomic alterations individually have small to negligible effects, collectively can change the physiology of a cancer through a variety of pathways. The most representative method is P-NET (Elmarakeby *et al.* 2021), a multi-layer hierarchical network based on child–parent relationships among encoded features, genes and pathways, which utilizes somatic mutations and copy number variations to predict the pathological stage (primary or metastatic) in prostate cancer patients. Compared to typical machine learning models, P-NET achieves better performance with a significantly reduced number of parameters, meanwhile improving the interpretability. In a similar spirit to DCell and P-NET, we have created a graph neural network in which units and parameters have a biophysical interpretation.

Pathway-based deep neural networks have shown that pathway can effectively extract features from molecular profiles. However, most models do not take into account the topological structure of pathways, and treat the pathway just as a set of genes. State-of-the-art methods such as P-NET take a step forward by exploiting the child–parent relationships between pathways. However, none of these methods take advantage of the parallel interactions between pathways, which contain hidden relationships between molecular features. In our opinion, a pathway represents a specific function, while multiple pathways are interconnected and influence each other to form a complex regulatory network. Therefore, the features of a pathway are not only determined by its member genes but also influenced by its associated pathways. In this paper, we fulfill the above idea in the proposed multi-head self-attention graph neural network to extract features from genomic profiles by using the pathway–pathway interaction network. GraphPath encodes each pathway as a neural unit, with sparse connections between neurons following pathway knowledge. A self-attention mechanism is used to capture the strength of relationships between pathways, and multiple heads represent different types of interactions between pathways. Compared to P-NET and other baseline methods, our model achieves better performance in all evaluation metrics. In addition to this robust performance, GraphPath’s predictions can also be explained by the neuron weights of specific pathways, allowing us to identify driver pathways. To our knowledge, this is the first practice that the pathway interaction network is embedded into a neural network to learn the latent distribution of the genomic profiling data.

## 2 Materials and methods

### 2.1 Data preparation

*Pathway data.* The architecture of GraphPath is founded upon two components: the gene-pathway membership annotation and the pathway–pathway interaction annotation, both of which are downloaded from the Kyoto Encyclopedia of Genes and Genomes (KEGG) PATHWAY database. KEGG is a manually curated resource integrating eighteen databases categorized into systems, genomic, chemical, and health information (Kanehisa *et al.* 2021). The PATHWAY database is the central database in KEGG, in which biological knowledge is captured from experimental data published in the literature, represented in terms of molecular interaction and reaction networks. A set of 511 pathways with the two types of annotations are collected from KEGG to build our model. The size of the gene set for these collected pathways (annotated as Pathsize) ranges from 2 to 1926, and the degree of the pathway interaction network ranges from 0 to 104.

*Cancer datasets.* In this study, we use the ‘Armenia *et al.*’ cohort (Armenia *et al.* 2018) as our benchmark dataset. This cohort comprises 1013 prostate cancer patients along with their corresponding copy number alterations (annotated in this study as CNA) and somatic mutations (annotated in this study as Mutation) data, which are prepared using a unified computational pipeline for harmonized somatic alteration derivation by P-NET [further details are available in the work (Elmarakeby *et al.* 2021)]. The benchmark dataset includes 333 cases of castration-resistant prostate cancer (CRPC) cases and 680 cases of primary prostate cancer. For external validation, we use two additional prostate cancer cohorts, one primary and one metastatic. More detailed information on these datasets can be found in Table 1.

**Table 1.** List of datasets with detailed information.

Type	Name	Genomic profiles (Number of genes)	Sample size (Metastatic, Primary)
Training and internal validation	‘Armenia *et al.*’ cohort	CNA (13 802), Mutation (15 741)	1013 (333, 680)
External validation	Metastatic	CNA (16 383), Mutation (16 383)	40 (40, 0)
	Primary	CNA (16 383), Mutation (15 005)	130 (0, 130)

### 2.2 Method overview

From a modern systems biology perspective, pathways are considered to be collectively involved in biological processes. Therefore, it is important to study the interactions between biological pathways to better understand the function of biological systems and to compensate for the limitations faced by traditional molecular models (Lu *et al.* 2007, Stoney *et al.* 2018).

**Figure 1.**
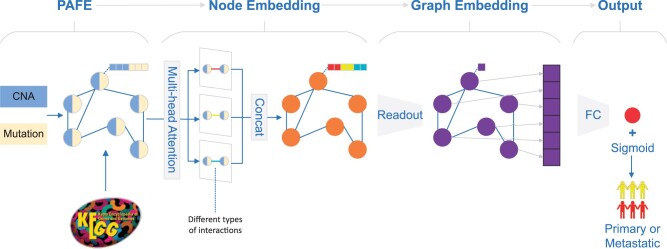
The framework of GraphPath. The workflow of the proposed model consists of pathway annotation-based feature extraction (PAFE), node embedding using a multi-head self-attention layer, graph embedding using a readout layer, and output prediction using a fully connected layer.

GraphPath mainly consists of four steps: (i) Pathway annotation-based feature extraction (PAFE), which is used to obtain the original features and the adjacency matrix of the pathway network; (ii) Multi-head self-attention based node embedding. The node (pathway) features are updated using a message-passing graph attention network (GAT) to aggregate the information of neighboring pathways; (iii) Graph embedding. We summarize the graph representation through computing a 1D vector score based on all node embeddings; (iv) Output. a fully connected layer is used to obtain the final binary (primary or metastatic) classification prediction. As a result, the molecular stratification problem is transformed into a graph classification task. The GraphPath workflow is shown in Fig. 1.

### 2.3 Pathway annotation-based feature extraction

First, for each omic data, the intersection of the gene set of that omic with the member gene set of all the pathways is obtained separately. This process identifies the genes that are common to both the omics and pathways. After the filtering process, there are 5004 genes in the CNA dataset and 7552 genes in the Mutation dataset. Then, the gene features of each omic of all the pathways are extracted according to the intersection of the corresponding omic. Finally, the gene features of multi-omics of pathways are concatenated to obtain the initial feature matrix of the pathway network. In existing methods, the pathway feature matrix is acquired based on a mask matrix, considering the pathway layer as a learnable hidden layer. In GraphPath, the pathway layer is input layer to extract pathway-level features from gene-level features.

Formally, for the given pathway graph G=(V,E), V indicate the node set, each node encoding a specific biological pathway; E indicate the edge set, each edge representing a known biological interaction between pathways. Initial node features $h=h_{1},\;h_{2},\ldots ,h_{N},h_{i}\in R^{F}$, where *N* is the total number of nodes (511), *F* is the dimension of a node initial feature vector (*F *=* *5004 + 7552). Specifically, *h*(*n*, *f)* = gene omic feature if the *f*th gene appears in the *n*th pathway, or *h*(*n*, *f*) = 0 otherwise. In addition, the adjacency matrix $A\in R^{n\times n}$ contain the graph structure information, where $A_{ij}=1$ means there is an edge between node $v_{i}$ and $v_{j}$, otherwise $A_{ij}=0$. In KEGG, pathway–pathway interactions are represented in each pathway map. For example, if a pathway appears at a certain location in another pathway’s map, this indicates that these two pathways interact with each other with respect to the activities or functions represented in that part of the map.

### 2.4 Multi-head self-attention based node embedding

In order to fully leverage the topological structure and initial features of the pathway network, we employ a multi-head self-attention integrated graph neural network for learning node embeddings. Graph convolutional network (GCN) has shown remarkable success in learning network graph structures from large-scale biological data (Zhang *et al.* 2017, Ahmed *et al.* 2020). To better incorporate the correlations between pathways into the model, we adopt the GAT, which enhances the feature learning power by introducing an attention mechanism. The GAT model acquires the discriminative feature of each node by calculating different influence weights of different neighbors, allowing for diverse interactions among input nodes, and utilizes multi-head to further enhance the representational capability of the model (Velickovic *et al.* 2017, Hu *et al.* 2021). This makes biological sense: on the one hand, there are different degrees of interactions between pathways, so a target pathway should receive different attention coefficients with different neighbors when generating the embedding for that pathway; on the other hand, to account for the effects of different types of interactions across pathways, the above procedure is repeated multiple times in parallel, implementing a multi-head mechanism.

Formally, node representations are updated by a weighted message passing scheme between neighbors, where the attention coefficient of node *j* to node *i* is formulated in the following equation:

(1) $$e_{ij}=\mathrm{softmax}(\mathrm{LeakyReLU}(a^{T}[Wh_{i}∥Wh_{j}]))$$

where $\cdot^{T}$ denotes matrix transposition; *a* represents the trainable node-level attention parameters for graph $G,\;a\in R^{2F^{\prime }};\;W$ is the trainable transformation weight matrix, $W\in R^{F\times F^{\prime }}$; ∥ denotes concatenation operation. The weights *a* and *W* are randomly initialized using the ‘Xavier’ initialization method, and optimized during the training process. We normalize the attention scores by applying softmax function. A multi-head attention mechanism is used to aggregate the 1-order neighbors of node *i* and the node itself (denotes as set $N_{i}$) to calculate its representation. More specifically, the self-attention is repeated *K* times independently, and then we concatenate the learned embeddings from each head to produce the output features for the node *i*:

(2) $${h^{\prime }}_{i}={∥}_{k=1}^{K}\sigma (\sum_{j\in N_{i}}e_{ij}^{k}W^{k}h_{j})$$

where $W^{k}\in R^{F\times F^{k}};\;K=3;\;\sigma (\cdot )$ is an activation function:

(3) $$\sigma =\mathrm{ELU}(x)=\{\begin{array}{cc} x, & x\;>0\\ \alpha (\text{exp}(x)-1), & x\leq 0 \end{array}$$

where *α* is a hyperparameter.

### 2.5 Graph embedding and output

The third layer is the readout layer. For summarizing graph representation from node embeddings, we use a sharing weight matrix to summarize a 1D vector representation for each node. The graph embedding *P* = $\left\{p_{1},\;p_{2},\;\ldots ,\;p_{N}\right\}$ is computed as follows:

(4) $$P_{i}=\mathrm{Tanh}(W^{p}h_{i}^{\prime })$$

where $W^{p}\in R^{F^{\prime \prime }}$.

In the last layer, a fully connected (FC) layer with a sigmoid function is used to generate the final prediction output:

(5) $$y=\mathrm{Sigmoid}(W^{y}P)$$

where $W^{y}\in R^{N}$. $W^{p}$ and $W^{y}$ are trainable.

Overall, the workflow of GraphPath includes pathway annotation-based feature extraction, node embedding using a multi-head self-attention layer, graph embedding using a readout layer, and output using a fully connected layer. GraphPath is end-to-end trainable, meaning that the model can learn to optimize the pathway embedding and classification prediction jointly.

### 2.6 Training and testing

In order to accurately distinguish the heterogeneity between primary and metastatic prostate cancer, we train and test GraphPath with the ‘Armenia *et al.*’ dataset. First, 30 random numbers are randomly generated, and then each method successively uses these 30 numbers as random seeds to divide the benchmark dataset into training set (80%), validation set (10%), and testing set (10%). The training set is used for training, the validation set is used to determine the early stopping criterion, and the testing set is used to assess model performance. Finally, the average of all the measures over 30 replications is taken as the final prediction performance. We use the SGD optimizer to reduce the binary cross-entropy loss function:

(6) $$\mathrm{BCELoss}=\frac{1}{N}\sum_{i=1}^{N}y_{j}*\log(p(\hat{y_{i}}))+(1-y_{i})*\log(1-p(\hat{y_{i}}))$$

where $y_{i}$ is the label for sample *i*; $p(\hat{y_{i}})$ is the prediction probability that sample *i* has metastatic cancer; *N* is the total number of samples. The initial learning rate is 0.05, dropout is 0.4, and weight decay is 0.05. The activation function of each hidden layer is the Tanh function:

(7) $$\mathrm{Tanh}=\frac{e^{2x}-1}{e^{2x}+1}$$

The activation function of the output layer is the sigmoid function:

(8) $$\mathrm{Sigmoid}=\frac{1}{1+e^{-x}}$$

## 3 Results

### 3.1 Performance metric

To evaluate the performance of the model, we use six evaluation metrics in this study, which are implemented using the scikit-learn library of Python: area under the receiver operating characteristic curve (AUC), area under the precision–recall curve (AUPR), F1 score (F1), Recall, Precision, and Accuracy. It is important to note that performance evaluation using these six metrics provides a comprehensive understanding of the model’s performance in classification tasks. These metrics are defined as follows:

(9) $$F1=\frac{2\times TP}{2\times TP+FP+FN}$$

(10) $$\mathrm{Recall}=\frac{TP}{TP+FN}$$

(11) $$\mathrm{Precision}=\frac{TP}{TP+FP}$$

(12) $$\mathrm{Accuracy}=\frac{TP+TN}{TP+TN+FP+FN}$$

where *TP*, *FP*, *TN* and *FN* are the number of true positives, false positives, true negatives and false negatives, respectively.

### 3.2 Comparison with baseline methods

The trained GraphPath outperforms P-NET on all six metrics (Table 2). In addition, we also compare GraphPath with self-constructed methods: a dense fully connected neural network without any restrictions (annotated as MLP) and a sparse nonfully connected network where the edges between the gene layer and the pathway layer are constructed based on the gene-pathway membership annotation (annotated as PAFE+MLP).

**Table 2.** Performance comparison of GraphPath with four methods based on the benchmark dataset.

Method	AUPR	AUC	F1	Recall	Precision	Accuracy
MLP	0.798 ± 0.066	0.872 ± 0.044	0.722 ± 0.067	0.704 ± 0.094	0.746 ± 0.058	0.821 ± 0.040
PPI-based	0.825 ± 0.058	0.899 ± 0.036	0.733 ± 0.078	0.701 ± 0.124	0.784 ± 0.072	0.833 ± 0.041
PAFE+MLP	0.863 ± 0.052	0.922 ± 0.031	0.777 ± 0.067	0.742 ± 0.091	0.823 ± 0.072	0.859 ± 0.040
P-NET	0.862 ± 0.052	0.905 ± 0.038	0.749 ± 0.054	0.671 ± 0.075	**0.857 ± 0.059**	0.851 ± 0.029
GraphPath	**0.887 ± 0.045**	**0.933 ± 0.028**	**0.808 ± 0.059**	**0.779 ± 0.074**	0.843 ± 0.070	**0.877 ± 0.038**

The best results are highlighted in bold.

In GraphPath, the pathway network is constructed based on biological interactions between pathways, but there are also other methods for constructing pathway networks (Francesconi *et al.* 2008, Liu *et al.* 2010, Xu *et al.* 2017). For example, protein-protein interaction (PPI) information is used to estimate the interactions between pathways. We take inspiration from PINTnet (Moon *et al.* 2017) to construct a PPI-based pathway network (annotated as PPI-based). In this method, an edge is created between two pathways if they have at least one overlapping gene, and there is at least one PPI between the overlapping gene(s) and the unique gene(s) of each pathway.

The experimental results indicate that GraphPath delivers superior performance over the currently available state-of-the-art pathway-based methods on all six evaluation metrics. Overall, these experimental results demonstrate that the pathway interaction network effectively captures and integrates the dominant part of each omics dataset, and GraphPath can be a valuable tool for accurately distinguishing the heterogeneity between different statuses of cancer patients. In addition, we performed *t*-test to test whether the metrics are significantly different between P-NET and GraphPath. The results are shown in Table 3.

**Table 3.** *P*-values of comparing GraphPath to P-NET for six metrics.

	AUPR	AUC	F1	Recall	Precision	Accuracy
P-NET versus GraphPath	0.0500	0.0036	0.0007	3.75E-06	0.4352	0.0104

### 3.3 Simulation experiment

We conducted simulation experiment following the methodology outlined in the work (Qu and Cui 2022). We first simulated a pathway network (pathway number = 800), based on which the R igraph package was used to depict relationships between nodes. We regarded the network structure as known prior knowledge, a variance-covariance matrix *M* was then defined according to the graph network information. To simulate pathway variables *X*, we generated a multivariate normal distribution with mean 0 and variance-covariance matrix *M*. We let 20 pathways have nonlinear effect:

(13) $$Y_{i}=\sum_{j=1}^{20}\left\{0.05X_{ij}^{2}+0.01X_{ij}^{3}+\text{exp}(-X_{ij}^{2}/10)\right\}$$

where $Y_{i}$ is the working response variable. By setting a threshold, we ultimately obtained 566 positive samples and 377 negative samples. After 30 rounds of Monte Carlo cross-validation, the following results in Table 4 are obtained. By comparing our model with MLP on the simulated graph dataset (annotated as simulation dataset), it is proved that our model can get better prediction results by using graph structure information. However, the hierarchy that P-NET relies on does not exist in the diagram, so it cannot be compared to P-NET. In addition, we performed *t*-test to test whether the metrics are significantly different between MLP and GraphPath, shown in Table 5.

**Table 4.** Performance comparison between GraphPath and MLP on simulation dataset.

Method	AUPR	AUC	F1	Recall	Precision	Accuracy
MLP	0.866 ± 0.037	0.819 ± 0.042	0.786 ± 0.039	0.785 ± 0.048	**0.789 ± 0.046**	0.744 ± 0.048
GraphPath	**0.890 ± 0.029**	**0.840 ± 0.036**	**0.811 ± 0.028**	**0.843 ± 0.038**	0.783 ± 0.036	**0.764 ± 0.037**

The best results are highlighted in bold.

**Table 5.** *P*-values of comparing GraphPath to MLP for six metrics.

	AUPR	AUC	F1	Recall	Precision	Accuracy
MLP versus GraphPath	0.0070	0.0016	6.16E-05	1.83E-09	0.2507	0.0031

### 3.4 External validation

Prediction models can only lead to improved outcomes for patient if they can guide healthcare professionals and individuals in making decisions about further management that are tailored to individual risk profiles, including additional testing, preventive interventions, lifestyle changes, monitoring, or treatment. That’s why it’s always important to test the generalizability of machine learning models on independent datasets to ensure their robustness and reliability. Generally, model predictive performance decreases in the validation dataset due to model over-fitting in the development cohort.

To further evaluate the generalizability of GraphPath, we perform external validations using two independent prostate cancer cohorts as inputs for the trained models, one primary (Fraser *et al.* 2017) (*n *=* *130) and one metastatic (Robinson *et al.* 2017) (*n *=* *40). We train our model on two balanced subsets (*N*: 333, *P*: 333) of the benchmark dataset, with no intersection of negative samples between subsets. The two independent datasets are merged into a single test set (*N*: 130, *P*: 40), which is fed separately into the two trained models to evaluate their performance. The prediction scores of the two models are averaged to produce the final predictions (Table 6). Our model achieves optimal performance on the two evaluation metrics—true-positive rate (TPR) and true-negative rate (TNR). The trained GraphPath model correctly classified 80.0% of the metastatic tumors and 79.6% of the primary tumors, indicating that the model can effectively generalize to unseen samples with stable and competitive discriminative performance.

**Table 6.** Performance comparison of GraphPath with four methods based on the external validation datasets.

Method	TPR	TNR
MLP	0.725	0.731
PAFE+MLP	0.725	0.746
PPI-based	0.800	0.739
P-NET	0.800	0.754
GraphPath	**0.800**	**0.796**

The best results are highlighted in bold.

### 3.5 Ablation experiment on multi-omics data

To assess the relative importance of specific molecular profiles contributing to the model prediction, we conduct ablation experiments based on three different input strategies (Table 7). The results show that removing each individual omics data leads to varying degrees of decrease in the evaluation metrics of the model classification. When using CNA data only, the model performance is reduced slightly. However, when using Mutation data only, all evaluation metrics are dropped significantly. These findings are consistent with previous reports: copy number variation is more informative compared to mutation (Hieronymus *et al.* 2014). The third input strategy, using both CNA and Mutation data, achieves the best performance, suggesting that the integration of these two omics data provides complementary information and improves overall prediction accuracy. This is consistent with the concept of multi-omics integration, which aims to capture a more comprehensive picture of the molecular landscape and enhance the predictive power of models. Overall, these ablation experiments highlight the importance of integrating multiple omics data types for accurate prediction and underscore the potential benefits of using GraphPath as a tool for multi-omics integration.

**Table 7.** Ablation experiments of GraphPath with three different input strategies.

	AUPR	AUC	F1	Recall	Precision	Accuracy
Mutation	0.609 ± 0.069	0.724 ± 0.086	0.381 ± 0.149	0.291 ± 0.125	0.581 ± 0.227	0.707 ± 0.036
CNA	0.866 ± 0.044	0.922 ± 0.022	0.788 ± 0.030	0.756 ± 0.044	0.829 ± 0.070	0.864 ± 0.023
ALL	**0.896 ± 0.043**	**0.948 ± 0.017**	**0.809 ± 0.054**	**0.774 ± 0.080**	**0.851 ± 0.041**	**0.879 ± 0.031**

The best results are highlighted in bold.

### 3.6 Graph embedding reveals cluster structure in t-SNE visualization

In order to visualize the classification performance and to explore the latent features extracted by GraphPath, t-distributed stochastic neighbor embedding (t-SNE) (Van der Maaten and Hinton 2008) dimension reduction is performed. t-SNE is a clustering and visualization method that has rapidly become a standard tool in a number of natural sciences. t-SNE works in three steps: First, the similarity between points in the higher-dimensional space is measured. Next, a distribution that measures the pairwise distances between points in the lower-dimensional embedding is calculated. Finally, KL divergence is used to minimize the difference between the probability distributions in the higher- and lower-dimensional spaces to produce the final 2D graph for visualization.

**Figure 2.**
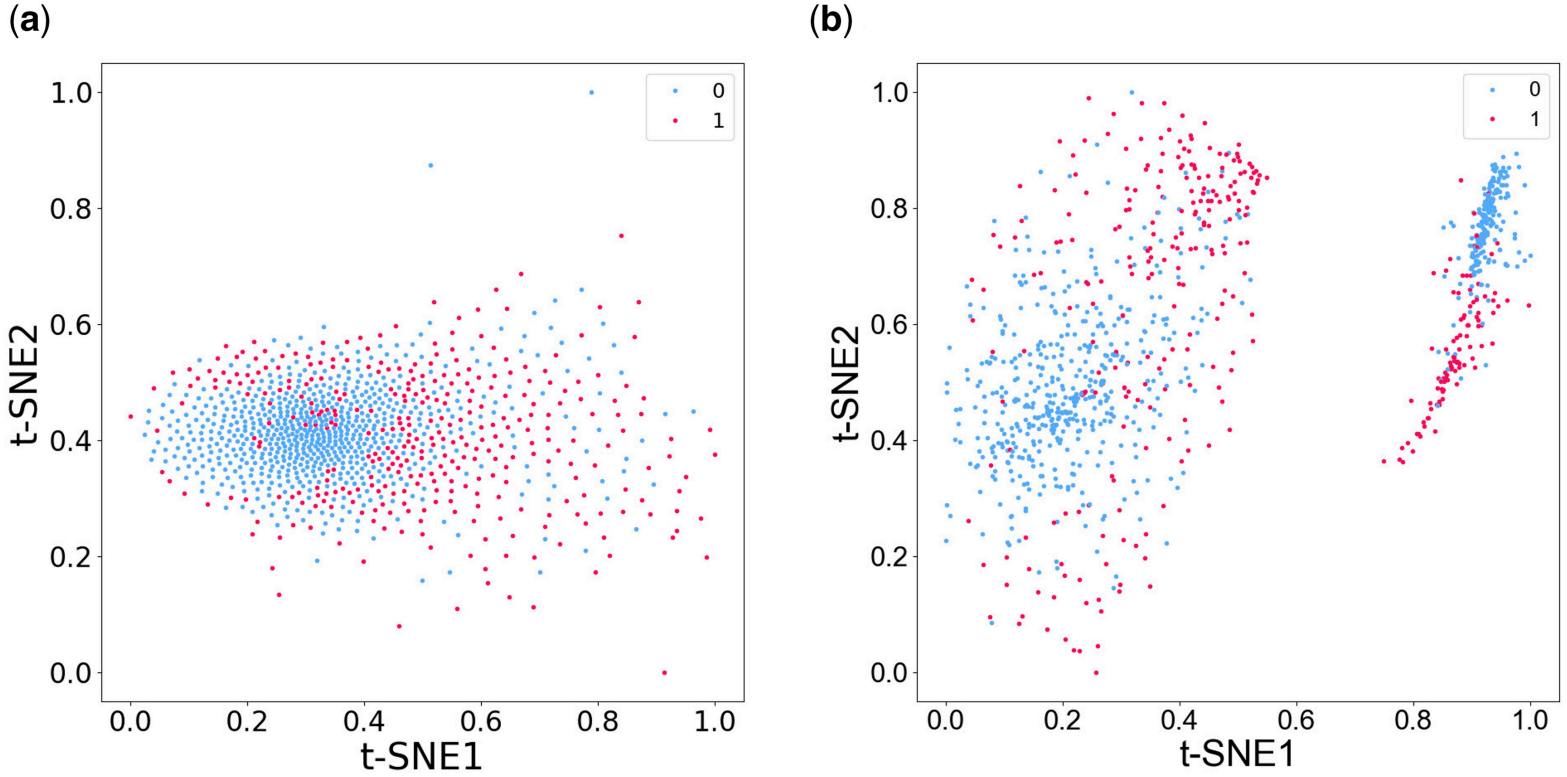
t-SNE visualization of (a) the pathway initial feature matrix and (b) the graph embedding generated by GraphPath.

In this study, we generate t-SNE plots on the pathway initial feature matrix and the graph embedding generated by GraphPath, shown in Fig. 2a and b, respectively. It can be seen that all of the samples are mixed together in Fig. 2a, illustrating that t-SNE cannot properly separate the initial features. In contrast, Fig. 2b demonstrates that the pathway network embedding can be effectively separated, proving that the model successfully learns a meaningful latent representation and reveals the meaningful neighborhood information contained in the graph embedding.

### 3.7 GraphPath identifies associated pathways and genes

**Figure 3.**
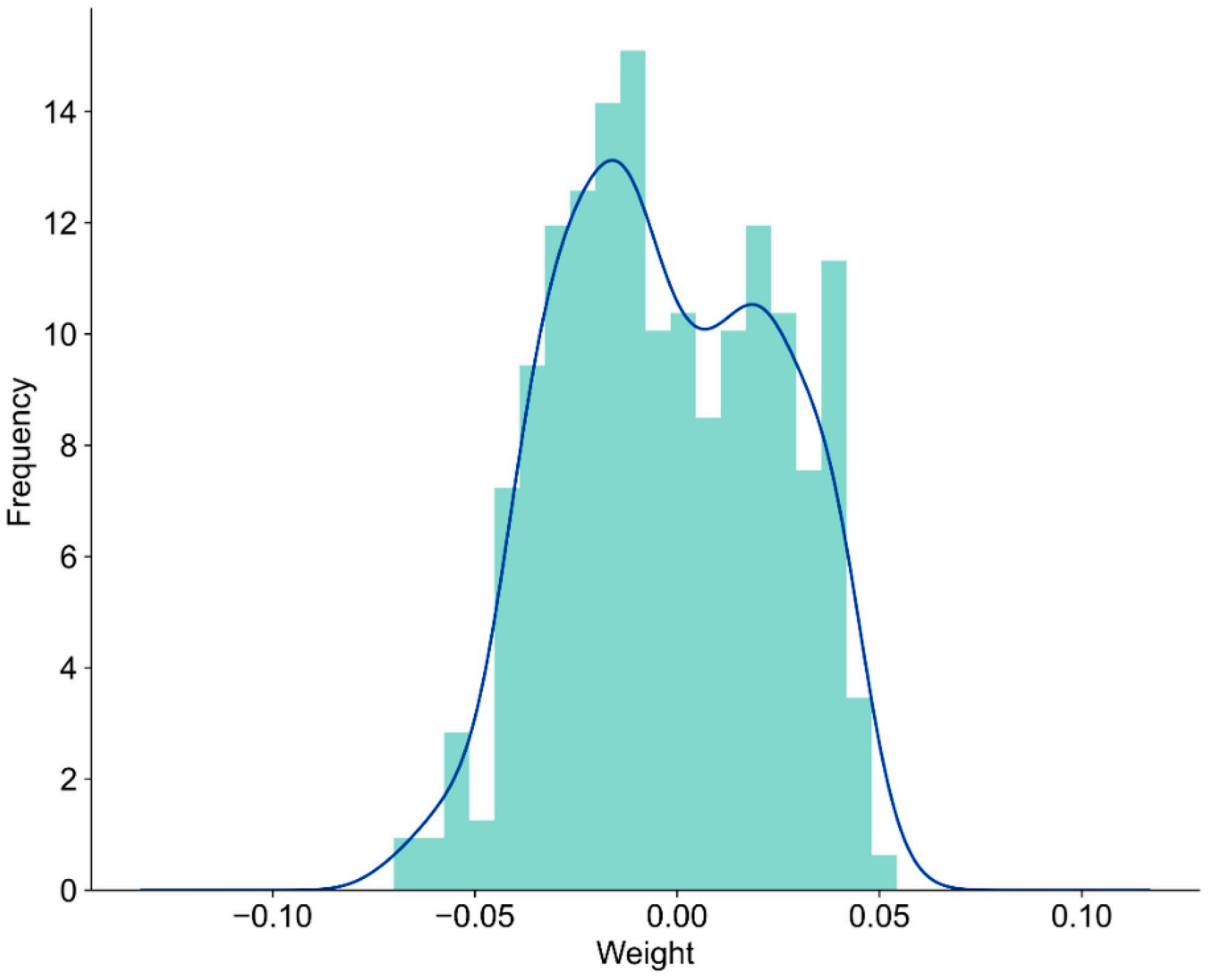
Weight distribution of the 511 pathways.

Although artificial neural networks are powerful classifiers, their internal structures are hard to interpret. In the life sciences, extensive knowledge of biology provides an opportunity to design visible neural networks (VNNs) that couple the model’s inner workings to those of real systems (Ma *et al.* 2018). GraphPath is a transparent neural network architecture in which the hidden nodes are constructed to physically correspond to biological units at a level of granularity helpful to human understanding, without recourse to any interpretation methods specialized for deep neural networks. We investigate which features are important for dependency prediction in GraphPath: when the final representation of each pathway is obtained in the ‘Graph Embedding’ layer, the prediction output can be obtained through a fully connected layer. Thus, we can identify pathways that are important for distinguishing cancer status by examining the weight of this FC layer. We visualize the weights for all pathways in the FC layer of GraphPath after training (Fig. 3). The top 10 pathways with the highest weights are selected (Table 8). The higher the weight, the greater the contribution of the pathway to the final prediction.

**Table 8.** The top 10 prostate cancer-related pathways inferred by GraphPath.

Rank	Pathway	Name	Evidence
1	ko05200	Pathway in cancer	
2	ko03000	Transcription factors	Pisano *et al.* (2021)
3	ko04131	Membrane trafficking	
4	ko05215	Prostate cancer	KEGG
5	ko05207	Chemical carcinogenesis-receptor activation	
6	ko03310	Nuclear receptors	Wang *et al.* (2021)
7	ko04147	Exosome	Akoto and Saini (2021)
8	ko03036	Chromosome and associated proteins	
9	ko04121	Ubiquitin System	Ummanni *et al.* (2011)
10	ko01001	Protein kinases	Shorning *et al.* (2020)

We find that pathways 1 and 5 are associated with cancers. Pathways 2, 4, 6, 7, 9, and 10 have been proven or reported to be associated with prostate cancer (Ummanni *et al.* 2011, Shorning *et al.* 2020, Akoto and Saini 2021, Pisano *et al.* 2021, Wang *et al.* 2021). For example, some ligand-independent members of nuclear receptor superfamily-designated as orphan nuclear receptors play significant roles in the growth regulation of prostate cancer via multiple AR-dependent or -independent pathways or mechanisms (Wang *et al.* 2021). In addition, intercellular communication is a key feature underlying prostate cancer progression and metastasis. There exists local signaling between prostate cancer cells and cells within the primary tumor microenvironment. Exosomes have been demonstrated to be involved in such signaling (Akoto and Saini 2021). It also makes sense that pathways 3 and 8 are highly weighted as their functions are widespread in cells. Moreover, pathway ko2000 with the largest Pathsize (1926) and pathway ko04010 with the largest degree (104) do not appear in the top 10 of the models, further demonstrating that GraphPath can find pathways that are truly associated with the target disease.

**Figure 4.**
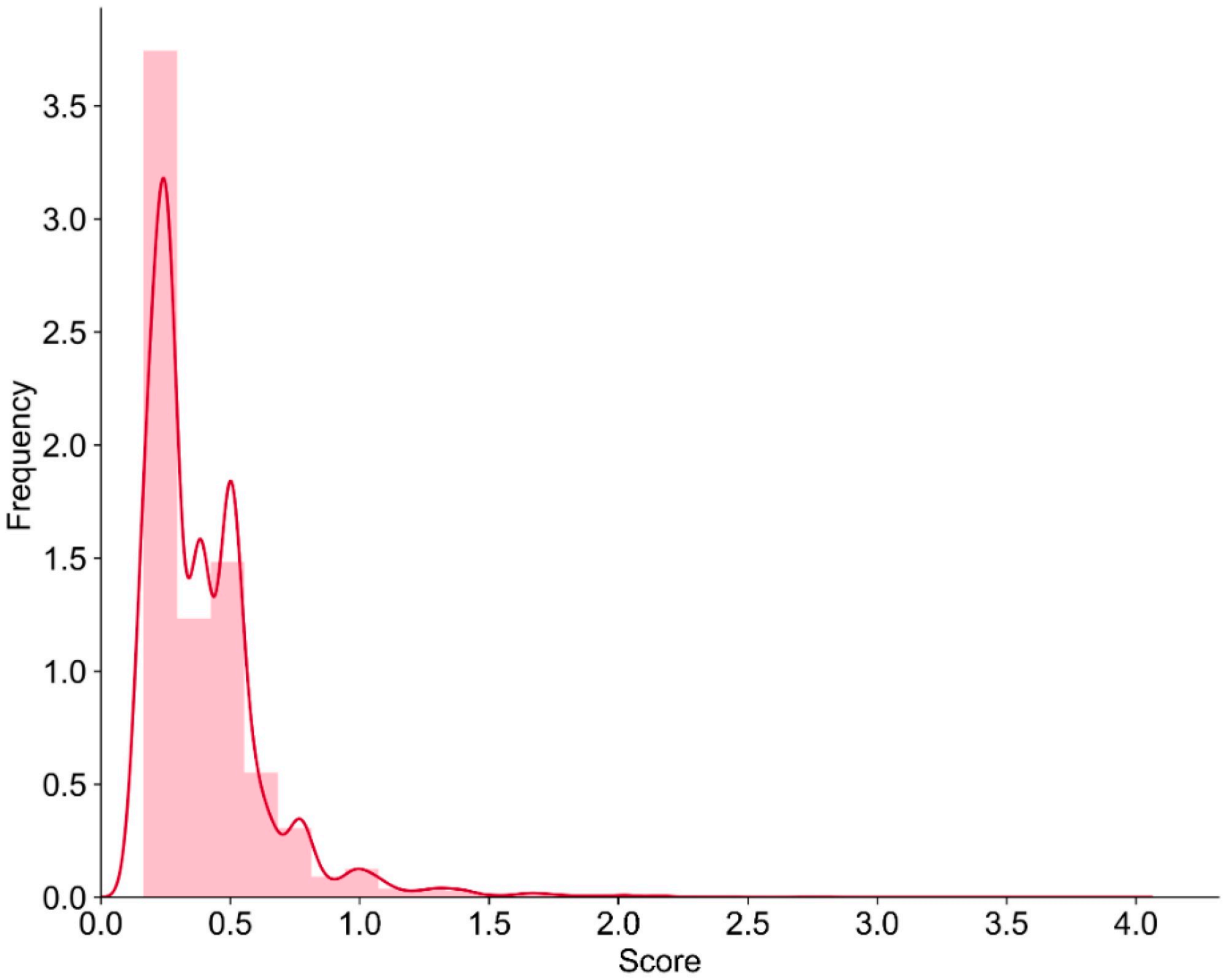
Contribution score distribution of the 6418 genes.

We further allocate pathway weights to each of its member genes. By summing the absolute values of the weights from different pathways, we obtain the final contribution scores for these genes. We analyze the contribution score distribution of all 6418 genes in the top 20 pathways (Fig. 4) and list the top 10 genes (Table 9).

**Table 9.** The top 10 prostate cancer-related genes inferred by GraphPath.

Rank	Gene name	Aliases	Evidence
1	ERK	EPH Receptor B2	Wang *et al.* (2018)
2	MTOR	Mechanistic Target of Rapamycin Kinase	Wang *et al.* (2018)
3	HRAS	HRas Proto-Oncogene, GTPase	
4	NRAS	NRAS Proto-Oncogene, GTPase	
5	KRAS	KRAS Proto-Oncogene, GTPase	Shtivelman *et al.* (2014)
6	NFKB1	Nuclear Factor Kappa B Subunit 1	
7	RELA	RELA Proto-Oncogene, NF-KB Subunit	
8	TP53	Tumor Protein P53	Wang *et al.* (2018)
9	EP300	E1A Binding Protein P300	
10	RAF1	Raf-1 Proto-Oncogene, Serine/Threonine Kinase	Shtivelman *et al.* (2014)

By searching the GeneCards database, we find that some of the highest-scoring genes, such as ERK, MTOR, TP53, and AR, have previously been reported as key genes associated with prostate cancer (Wang *et al.* 2018). Other unlisted high-scoring genes, such as BRCA1 and BRCA2, have been confirmed to be involved in DNA repair and are also linked to prostate cancer (Sathianathen *et al.* 2018). The identification of these genes as important by GraphPath further supports their relevance to prostate cancer. The distribution of scores across all 6418 genes appears to follow a power-law distribution, with a few highly important genes and many less important ones. This is a common observation in biological networks and highlights the importance of identifying key nodes in these complex systems.

In summary, the pathways and genes identified by GraphPath contribute to understanding the mechanisms underlying tumor progression and metastasis. Moreover, we believe that our method will help drive the development of targeted drug therapies for cancer treatment.

## 4 Discussion and conclusion

Cancers that share similar morphological characteristics often exhibit distinct treatment responses and prognoses. While molecular biology focuses on the impact of individual genes on the cancer state, functional genomics assesses comprehensive genetic alterations in cancer cells and seeks to integrate dynamic changes in these networks to explain cancer phenotypes. Our research focuses on exploring how pathway knowledge can be used to design deep neural networks for cancer prediction and interpretation, and thus we develop GraphPath. We demonstrate that GraphPath achieves superior predictive performance in the task of cancer molecular stratification and provides interpretable predictions. Our work also highlights the importance of integrating systems biology knowledge into the architecture of graph neural networks.

Although the prior knowledge of pathways is reliable, there is a high degree of redundancy between different pathway databases, and the ways in which pathways are created and named are inconsistencies, lacking a unified standard. However, with the ongoing advancement of pathway research, we believe that the pathway interaction network will become as important as the PPI networks and gene regulatory networks in the study of biological regulation. In other words, the pathway network will play a crucial role in understanding the complex interactions and functions of biological systems, and can be widely applied. The model design idea proposed in this paper will become increasingly valuable as the field develops.

Overall, GraphPath is a biological knowledge-driven graph neural network that encodes the pathway–pathway interaction network and achieves optimal performance in stratifying cancer status. Our approach provides a promising framework for systems biology to improve the understanding of biological processes relevant to disease. By providing an interpretable algorithm, GraphPath has the potential to identify new biomarkers and potential drug targets.

## Data Availability

All datasets utilized in this study are publicly available, and the source code is accessible online at https://github.com/amazingma/GraphPath.
